# Arabic Reading Performance With a Chromatic Acuity Chart

**DOI:** 10.1167/iovs.66.4.3

**Published:** 2025-04-01

**Authors:** Balsam Alabdulkader, Ali Almustanyir, Norah Alsalem, Essam Almutleb, Mosaad Alhassan, Jeffery K. Hovis

**Affiliations:** 1 Optometry and Vision Sciences, Applied Medical Sciences College, King Saud University, Riyadh, Saudi Arabia; 2 Optometry and Vision Sciences, University of Waterloo, Waterloo, Ontario, Canada

**Keywords:** reading performance, Balsam Alabdulkader-Leat chart, chromatic chart, color vision deficiency

## Abstract

**Purpose:**

This study compared the reading performance for Arabic text defined by chromatic and achromatic contrast to understand better how chromatic contrast affects reading of normally sighted individuals and to establish a baseline for determining whether patients have a selective red–green chromatic sensitivity loss.

**Method:**

Reading performance for Arabic text was accessed by examining maximum reading speed (MRS), reading acuity (RA), critical print size (CPS), and the Reading Accessibility Index (ACC) using three near-point charts. The charts were the black-on-white Balsam Alabdulkader–Leat (BAL) chart, a red-on-green chart, and a gray-on-gray chart with a background luminance equal to the chromatic chart.

**Results:**

The MRSs were significantly different (*P* = 0.03), with the red-on-green chart having a slightly higher value than the BAL chart. The ACC was lower for the BAL chart than the red-on-green and gray charts (*P* = 0.003). However, RA for the BAL chart was better, and the CPS was smaller relative to the red-on-green chart (*P* < 0.05) and gray chart (*P* < 0.001). Individuals with red–green color vision deficiencies had poorer RA and larger CPS on the red-on-green chart relative to the achromatic charts.

**Conclusions:**

Although the MRS and ACC of the chromatic chart were significantly higher, the difference was not clinically important. The result that the MRS was similar for all three charts confirmed earlier findings that MRS is similar if text contrast is sufficiently above threshold. The lower RA and corresponding larger CPS for the red-on-green and gray charts were due to their lower background luminance and lower contrast.

Reading is a vital skill that plays a pivotal role in the functioning of individuals within a literate society. Numerous factors contribute to one's ability to read proficiently. These factors can be broadly classified into two groups: those that relate to the individual reader and those associated with the reading material.^1^ The reader-related factors encompass elements such as their education level and level of interest in the material at hand. The education level is a significant determinant, as it often dictates the reader's skill level and comprehension capability. Likewise, the reader's engagement and interest in the material can considerably sway their reading efficiency and retention. The reading material (stimulus)–related factors include parameters such as the size and color of the text, the luminance and color contrast between the text and the background, and the difficulty level of the content (including whether the stimuli are letters, words, or continuous text).^1^^–^^3^ These stimuli are vital to the readability of the content, as they affect the reader's ability to process and understand the information presented.^1^^,^^2^

Researchers have delved into the intriguing relationship between color and reading for decades, investigating how color can affect reading performance.^3^^–^^5^ Manipulating luminance and/or luminance contrast and chromatic contrast has been a key area of study for enhancing reading performance and perceptual abilities, especially in individuals with impaired vision.^3^^,^^4^^,^^6^ Altering the lighting and color for reading has been demonstrated to significantly improve visual comfort, which is particularly beneficial for individuals with poor vision.^3^^,^^5^ These adjustments can mitigate common perceptual difficulties, allowing for better reading performance with less eye strain and increased focus.^7^^,^^8^ In many existing studies on reading performance, changes to luminance and chromatic contrast are often introduced simultaneously,^4^^,^^5^^,^^9^ complicating the assessment of their individual effects on performance. This overlap limits the ability to attribute improvements specifically to luminance or to chromatic contrast. Therefore, it is essential for future research to distinguish between these factors to evaluate their distinct contributions accurately.

With regard to investigating reading performance for text defined by only chromatic contrast, the studies are few. Legge et al.^4^ reported that, for text displayed against backgrounds of equal or nearly equal luminance, the maximum reading speeds (MRSs) achieved with chromatically distinct text by individuals with normal vision, which is well above threshold levels in size, were comparable to those attained with text defined solely by luminance contrast. When expressed in multiples of a threshold value, the reading speed versus contrast functions for achromatic and chromatic letters superimposed.^4^ However, Legge et al.^4^ also reported that, in contrast to normally sighted subjects, individuals with low vision from various causes had reduced reading performance for isoluminant red–green text and often never reached the MRS of the achromatic text as the chromatic contrast increased.^4^

Other studies^3^^,^^10^^,^^11^ have examined how adding chromatic contrast to luminous contrast affects reading performance and text legibility. Knoblauch et al.^3^ noted that, when achromatic contrast exceeded a 12% Michelson threshold, adding chromatic information did not bolster reading performance in visually normal participants. Conversely, including chromatic contrast to text with lower achromatic contrast enhanced reading speed, although not to the level observed with the high-contrast achromatic text. McLean^10^ showed similar results in reading instrument displays; however, adding chromatic contrast was more effective for text with a positive achromatic (brighter text) contrast. Penkelink and Besuijen^11^ reported that legibility and perceived contrast improved when chromatic contrast increased for isoluminant text, but increases in chromatic contrast did not improve these subjective ratings if the Michaelson luminous contrast was 50% (the lowest luminous contrast used in their experiment) or higher, consistent with the findings of Knoblauch et al.^3^

The loss in reading performance for chromatically defined text for low-vision subjects indicates that text using chromatic contrast could play an important role in clinical diagnostics and may contribute to understanding how chromatic contrast can enhance reading performance.^3^^,^^4^^,^^6^ To assess reading performance with chromatically defined text, one could standardize the letter size and measure performance across various levels of chromatic contrast, employing either calibrated computer monitors with custom software or a range of printed materials with systematically altered chromatic contrasts. However, these methods may pose challenges in many clinical settings due to the required time commitment and equipment cost. By comparison, this study employed a methodology that keeps chromatic contrast constant while varying the text size in descending order. This approach enables a direct comparison with the reading performance associated with achromatic text. For this purpose, a new Arabic reading performance chart was created, featuring red text on a green background. The design ensures that the text and background have approximately equal luminance while providing a sufficient chromatic contrast along the red–green chromatic axis.

This study built on previous work in developing a chromatically defined near-point chart to measure Arabic reading performance by testing a larger sample of individuals with normal vision to validate the results further.^12^^,^^13^ Previous work reported preliminary findings that maximum reading rates for sufficiently large (exceeding threshold size) chromatic text paralleled those for high-contrast achromatic text.^13^ This study also addressed the limitation of the previous work where the luminance disparity between the backgrounds of the achromatic and chromatic texts may have influenced the reading performance outcomes. This investigation compared Arabic reading performance found with chromatically defined text on a colored background with that found using achromatic text against a gray background with luminance equal to that of the green background of the chromatic chart. Additionally, in this study, the luminance contrast of the characters on the achromatic gray chart was set to yield reading acuity and critical print size akin to those available on the chromatic chart, thus providing a more nuanced understanding of the impact of these variables on reading performance. Arabic reading performance was also determined for the original black print on a white background chart to confirm the previous findings using a larger group of participants.

The red–green chromatic contrast was selected over other possible chromatic discrimination axes because the earlier study by Legge et al.^4^ provided sufficient information for setting our achromatic and chromatic contrast levels (see Methods). In addition to their results showing that individuals with low vision have a greater loss in reading performance for red–green text, other studies^14^^,^^15^ have reported that some individuals with cone disorders, diabetic retinopathy, and optic atrophy have a reduced reading rate for red text on a dark background, further suggesting that measuring reading performance text defined by red–green chromatic contrast may be helpful in assessing and managing individuals with low vision.

To quantitatively assess reading capabilities for the different charts, the measurement of reading performance is utilized as a gold standard method.^1^ This includes parameters such as MRS (in standard words per minute [WPM]), reading acuity (RA, in logMAR, where smaller numbers are better), critical print size (CPS, in logMAR, again where smaller numbers are better), and the Reading Accessibility Index (ACC), which is a ratio ranging from 0 (poor) to ∼1.0 (average) to >1.0 (excellent).^16^

## Methods

Following the pilot study,^13^ a subsequent exploratory investigation was designed to further examine the effect of colored text on Arabic contextual reading performance. The objective of the previously developed chart was to study the effect of color contrast on reading performance. It was constructed of red text on a green background; the letters and the background had approximately the same luminance but differed in chromaticity (color contrast). The methodology for developing the red-on-green chart was detailed in the previously published pilot study.^13^ Briefly, the colors were chosen so that they were in the red–green region of the chromaticity diagram where the deuteranopic and protanopic lines of confusion were close together, so it was possible to detect both types of defects with just two colors.^17^ This reduced the S-cone contribution to the chromatic contrast and potentially minimized the contribution of short wavelengths to the chromatic aberration between images formed by the medium and long visible wavelengths. The L-/M-cone contrast was selected so that it was near the plateau of the reading speed–print size function for larger print based on the findings by Legge et al.^4^ for the 1° letter height. In addition, the colors had to be within the standardized Pantone color system. The chromaticity coordinates and luminances were measured under standard lighting conditions (G45-14 E27; NewPower Lighting, Sharjah, UAE; color rendering index > 90; correlated color temperature, 6500K) using a CS-100A Luminance and Color Meter (Konica Minolta, Tokyo, Japan). The same light source and conditions were used in the reading performance trials. The final Pantone colors were then sent to Precision Vision (Woodstock, IL, USA) for professional printing.

**Figure 1.**
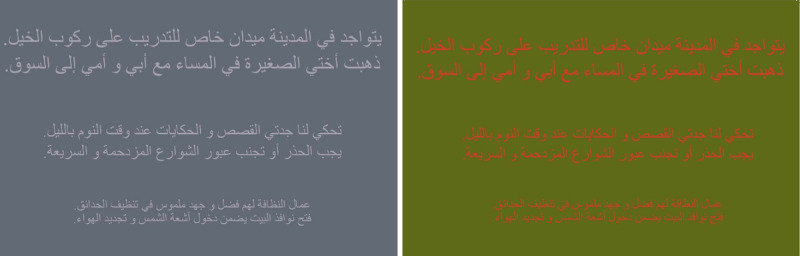
Gray BAL chart (*left*) and red-on-green BAL chart (*right*).

The gray control version of the Balsam Alabdulkader–Leat (BAL) chart used the same text characteristics and progression of sizes as the chromatic and original BAL charts. The primary objective was to have a reading chart that had the same background luminance as the chromatic chart with a Michelson luminous contrast that would result in a similar reading performance as the chromatic chart for normal participants. The target contrast value was approximately 30% so that the reading performance would be similar to the performance on the chromatic chart.^4^ In addition to equating the background luminance, this chart would provide a better reference for evaluating potential luminous contrast artifacts that may be present in the red-on-green chart due to printing nonuniformities or chromatic aberration. After measuring and selecting the gray chart colors with the CS-100A meter, the specifications of the charts were then printed by Precision Vision. Figure 1 shows the two new charts used in this study.

Using the previously described lamp, the illuminance on the tests was set at 300 lux in the plane of the chart, the background luminance of the printed gray and red-on-green charts was 13.6 cd/m^2^, and the background luminance for the original (black-white) BAL chart was 95 cd/m^2^. The Michelson luminous contrast for the achromatic (black-on-white) original BAL chart was 100% (Weber contrast 100%), whereas the luminous contrast of the chromatic (red-on-green) BAL chart was 0.8% (Weber contrast 1.6%) and for the gray chart it was 28% (Weber contrast 77.8%).

We were unable to independently measure both the chromaticities and luminances of the text and background; however, the printer's specified tolerance for the luminous contrast in the charts was maintained to be within a ±5% Michelson contrast range, and the color tolerance was ΔE ab * = 3. We measured the background luminances of the gray and red–green charts at 12 different locations using the CS-100A meter and light source. Based on 2 SD, the luminance uniformity was ±2.3% for both charts. The average ΔE^*^_ab_ between the locations of the green background was 1.74 ± 0.94. Using an average ΔE ab * value below 2.2 as threshold,^18^ the nonuniformities in the background color would be undetectable.

### Participants

The 94 participants were recruited from the student and staff population at King Saud University in Riyadh, all of whom had a minimum education level of a high school diploma. The inclusion criteria were near visual acuity < 0.1 logMAR, fluency in reading and speaking Arabic, and normal color vision as determined by history or the Ishihara test if there was any uncertainty. Near visual acuity was measured at 40 cm using the Tumbling E Early Treatment Diabetic Retinopathy Study (ETDRS) near chart. Only 38% of participants had refractive error and wore their usual refractive correction, primarily glasses, or contact lenses. Although chromatic aberration could be a concern with higher Abbe value lenses, the majority of our participants either had no refractive error or low to moderate prescriptions (e.g., within ±2.00 diopters). Thus, the impact of chromatic aberration on our findings was likely minimal. Individuals were excluded if they had any difficulties or reported developmental delays that may have affected their reading results.

In addition to the main study, four individuals with congenital red–green color vision defects were included. The primary reason for including these individuals was to determine if there were luminance contrast artifacts on the red-on-green chart that could influence reading performance. The artifacts could be due to the printing process, chromatic aberration, or both. If the reading performance of these individuals, especially individuals with more severe defects, fell within the normal range, then this could indicate that there were significant luminance contrast artifacts in the red–green color chart. Based on the anomaloscope results, this group consisted of one mild deuteranomaly, one moderate deuteranomaly, one deuteranope, and one mild protanomaly.

The research procedure complied with the tenets of the Declaration of Helsinki and received approval from the Ethics Committee of King Saud University (Project No. E-21-6309). All participants provided their informed consent before participating in the study.

### Procedure

Arabic reading performance was assessed using the original black-on-white (original) chart,^11^ the red-on-green chart, and the gray chart. The three charts have different sets of sentences but have the same difficulty level.^19^ The order of the charts was chosen randomly for each participant. Reading performance tests were carried out binocularly while participant wore their habitual correction. Each reading session commenced with a demonstration trial, utilizing a series of demonstration sentences to acquaint participants with the testing protocol. A wooden reading stand ensured a consistent reading distance of 40 cm throughout the test. The previously described lamp was used to illuminate the cards at 300 lux, and the ambient illumination surrounding the stand was set to 400 lux in the plane parallel to the table where the stand was placed.^20^ Each chart was covered to prevent participants from seeing it before the test.^12^ The examiner unveiled the chart, instructing participants to read aloud as quickly and accurately as possible without correcting any mistakes.^1^^,^^12^ All reading sessions were audiorecorded to facilitate the post-session calculation of error counts for each sentence and the precise measurement of reading speed.^12^

### Reading Performance Data

The primary outcome measures included MRS in standard-length words per minute (SLWPM), RA, CPS, and the ACC. A comprehensive description of these reading performance measures for Arabic has been provided in other publications.^12^^,^^19^^,^^21^ The MRS and RA for SLWPM were computed using the following equation^1^^,^^12^:

$$\begin{array}{ccc} & & MRS\left(SLWPM\right)\;=\;60\;\\ & & \;\times \;\left[\frac{\left(\mathrm{Number}\;\mathrm{of}\;\mathrm{standard}\;\mathrm{words}\;-\;\left(\frac{\mathrm{Number}\;\mathrm{of}\;\mathrm{errors}\;\mathrm{in}\;\mathrm{characters}}{5}\right)\right)}{\mathrm{Time}\;\mathrm{in}\;\mathrm{seconds}}\right] \end{array}$$

$$\begin{array}{ccc} & & RA\left(logMAR\right)=Smallest\;print\;size\;attempted\;\\ & & \;+\left[\left(\frac{\mathrm{Numbers}\;\mathrm{of}\;\mathrm{errors}\;\mathrm{in}\;\mathrm{characters}}{5}\right)\;\times \;0.005\right] \end{array}$$

CPS was defined as the print size in logMAR units that resulted in 80% of the average reading speed of the plateau. The ACC was calculated as follows^16^:

$$\begin{array}{ccc} & & ACC\\ & & \;=\frac{\mathrm{Average}\;\mathrm{of}\;\mathrm{the}\;\mathrm{reading}\;\mathrm{speed}\;\mathrm{of}\;\mathrm{the}\;\mathrm{largest}\;8\;\mathrm{print}\;\mathrm{sizes}}{175} \end{array}$$

### Statistical Analysis

The normality of the data was evaluated using the Kolmogorov–Smirnov test. The test results indicated that both the log and linear MRS and ACC data were normally distributed (*P* > 0.1) for all charts. Conversely, the data for RA and CPS did not follow a normal distribution for gray and red-on-green charts (*P* < 0.05) but was normally distributed for the original chart (*P* > 0.1).

The differences in reading speed and ACC were examined using one-way repeated-measures ANOVA. To further investigate these differences, post hoc analyses were carried out using the Tukey test, and the Friedman test with Dunn's post hoc test was used for non-parametric analyses of the RA and CPS.

A post hoc power analysis using G*Power was used to evaluate the statistical power of our study. The analysis was based on an observed Cohen's *d* of 1.78, reflecting a large effect size between the two groups in RA (group 1, –0.15; group 2, 0.01). With a total sample size of 94 and an alpha level set at 0.05, our study achieved a power of 0.96, sufficient to detect significant differences between the groups. Data were analyzed using Prism 10.0.3 for Windows (GraphPad, Boston, MA, USA).

## Results

The participants ranged in age from 20 to 38 years (mean ± SD, 23 ± 4.7). The average near visual acuity was –0.07 ± 0.09 logMAR, ranging from –0.30 to 0.09 logMAR.

### Reading Performance Outcomes

The mean MRSs and ACCs across the three charts are summarized in Table 1. Statistical analyses revealed a significant difference among the charts in the logarithmic transformation of the data for MRS (log-SLWPM), *F* (1.873, 172.3) = 3.55, *P* = 0.03. Tukey's post hoc analysis indicated that the reading speed for the red-on-green chart (2.254 log-SLWPM) was significantly higher than that for the original chart (2.246 log-SLWPM) (*q* = 4.22, *P* = 0.01), but the other comparisons were not statistically significant. However, despite being statistically significant, this difference is not clinically important, as it is a difference of less than 10 WPM.^22^ Similar results were observed for the untransformed MRS results (SLWPM). The ANOVA showed a significant difference among the charts, *F* (1.884, 173.4) = 4.45, *P* = 0.01, where the reading speed for the red-on-green chart (182.6 WPM) was significantly higher than that for the original chart (178.7 WPM) (*q* = 4.70, *P* = 0.00), but the other comparisons were not statistically significant.

**Table 1.** MRS, RA, CPS, and ACC for Each Arabic Reading Chart and the 94 Participants

	Original Chart (Black-on-White)	Red-on-Green Chart	Gray Chart
MRS (SLWPM)			
Mean ± SD	178.7 ± 24.88	182.6 ± 25.75^*^	180.4 ± 25.20
Median	177.3	183.2	181.7
Range	127.0–277.2	130.5–268.1	122.7–250.5
MRS (log-SLWPM)			
Mean ± SD	2.246 ± 0.06	2.254 ± 0.06^*^	2.250 ± 0.06
Median	2.249	2.260	2.256
Range	2.09–2.44	2.11–2.42	2.08–2.39
RA (logMAR)			
Mean ± SD	−0.15 ± 0.09	0.01 ± 0.09^*^	0.01 ± 0.08^†^
Median	−0.15	0.00	−0.01
Range	−0.29 to 0.16	−0.16 to 0.35	−0.13 to 0.34
CPS (logMAR)			
Mean ± SD	0.05 ± 0.14	0.22 ± 0.13^*^	0.21 ± 0.10^†^
Median	0.10	0.20	0.20
Range	−0.20 to 0.50	−0.10 to 0.60	−0.10 to 0.50
ACC			
Mean ± SD	1.04 ± 0.14	1.06 ± 0.15^*^	1.06 ± 0.15^†^
Median	1.04	1.07	1.06
Range	0.73–1.54	0.78–1.60	0.73–1.50

* Significant difference between original chart and red-on-green chart (*P* < 0.05).

† Significant difference between original chart and gray chart (*P* < 0.05).

In line with the reading speed outcomes, the ANOVA identified a significant difference in the ACC across the three charts, *F*(1.968, 181.0) = 5.73, *P* = 0.004. The Tukey test showed that the ACC was significantly lower (worse) for the original chart compared to both the red-on-green and gray charts (*q* = 4.7, *P* = 0.003 and *q* = 3.7, *P* = 0.02, respectively).

The Friedman test was conducted to evaluate the differences in the CPS across the three charts. The results showed a significant difference among the charts (χ^2^ [2 df] = 118, *P* < 0.05). Dunn's post hoc test revealed that the CPS for the original high-contrast black-on-white chart was significantly smaller (better) than both the red-on-green chart (*z* = 8.25, *P* < 0.001) and the gray chart (*z* = 7.81, *P* < 0.001). However, because of design decisions, no significant difference was found between the red-on-green chart and the gray chart (*z* = 0.44, *P* > 0.99), as expected.

The Friedman test (χ^2^ [2 df] = 152.1, *P* < 0.001) also showed a significant difference in RA across the three reading charts. Similar to the CPS results, Dunn's post hoc test showed that RA with the original high contrast black-on-white chart was significantly better (i.e., smaller values denote improved performance) compared to the red-on-green and gray charts (*z* = 10.34, *P* < 0.001, and *z* = 10.12, *P* < 0.001, respectively). The difference in the mean reading acuities between the original chart and both the red-on-green and gray charts was around 1.5 lines, a clinically important difference.^23^

**Figure 2.**
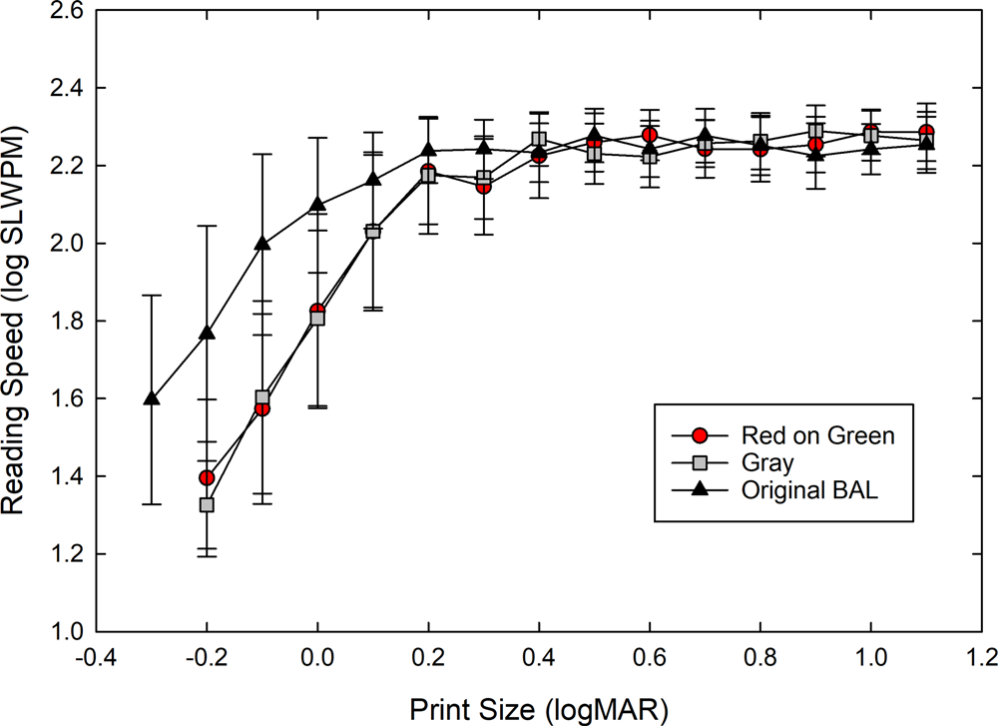
Comparison of average reading speeds for different print sizes across black-on-white (original), red-on-green, and gray reading charts. Error bars are ±1 SD.

To further examine the differences in reading performance across the three charts, the average reading speeds for each print size were plotted as a function of print size, as shown in Figure 2. The results show the expected typical reading speed curve for the original chart, with reading speed increasing as a function of print size until reaching a plateau for the larger fonts.^12^^,^^21^ The gray and red-on-green charts show a similar trend, but the curves are shifted toward the larger print sizes, which is expected given the lower luminance of both charts (14 cd/m^2^ compared to 95 cd/m^2^) and their lower luminance and chromatic contrasts.

### Results for Color-Deficient Participants

Table 2 summarizes the major findings of the color-defective participants. Similar to the color-normal participants, the MRS is essentially the same for all three charts. Also, the RA for the gray chart was lower and the CPS was larger (worse) than those of the original chart. The differences were a lower RA on the red-on-green chart relative to the other two achromatic charts, and the CPS for the red-on-green chart was larger (worse) than the performance on the gray chart for the two individuals with the more severe color vision defects.

**Table 2.** Summary of Results for the Color-Defective Participants for MRS, RA, CPS, and ACC for the Three Charts

	Original Chart (Black-on-White)	Red-on-Green Chart	Gray Chart
MRS (log-SLWPM)			
Mild PA (*n* = 1)	2.30	2.29	2.30
Mild DA (*n* = 1)	2.17	2.20	2.23
DA (*n* = 1)	2.41	2.41	2.34
D (*n* = 1)	2.28	2.25	2.21
RA (logMAR)			
Mild PA	−0.17	−0.023	−0.046
Mild DA	−0.20	0.11	0.028
DA	−0.28	−0.024	−0.047
D	−0.19	0.12	−0.039
CPS (logMAR)			
Mild PA	−0.14	0.16	0.18
Mild DA	0.094	0.11	0.13
DA	−0.12	0.16	0.084
D	−0.01	0.19	0.039

D, deuteranopia; DA, moderate deuteranomaly; PA, mild protanomaly.

## Discussion

The primary goal of this project was to establish norms for a chromatically defined near reading chart using the Arabic script. The chart was designed to be a clinical application of the research by Legge et al.^4^ using chromatically defined text. To our knowledge, this type of chart has not been used before. Another goal was to compare these data with the reading performance found using a gray chart with approximately the same background luminance and a contrast designed to produce a similar reading performance as the red-on-green chart. This latter property would provide a better baseline for distinguishing between a selective loss in red–green chromatic channels versus a more general loss in both the achromatic and red–green chromatic channels if the reference was the original black-on-white high contrast chart with the brighter background (13.6 cd/m^2^ compared to 95 cd/m^2^).

The results for the color-normal participants reflect the expected finding that the MRS would be similar across the charts when the print size was sufficiently large to counteract the lower luminances and lower contrasts of the gray chart and the red-on-green chart.^4^ Because the ACC is based on data generated at the larger print sizes, it is also similar across the three charts. The major differences are the lower RA and corresponding larger (worse) CPS for the gray and red-on-green charts relative to the original high-contrast black-on-white chart. These differences result from the lower luminance/lower contrast of the gray and red-on-green charts.^4^^,^^15^ Provided that such a change is sufficient to reduce the RA, lowering the luminance and/or contrast essentially shifts the reading speed versus print size curve horizontally to larger letters.^24^^–^^26^ That these two values were similar for the gray chart and the red-on-green chart indicates that the design goal of having an achromatic chart of equivalent luminous contrast (based on reading performance) and luminous reflectance as the red-on-green chart was achieved. Thus, any significant differences in performance between the gray and red-on-green acuities found for observers in a clinical setting could be attributed to differences in luminous and chromatic contrast sensitivity without being confounded by any luminance difference, which is present if the red-on-green is compared to the original chart.

Nevertheless, the similarity in the reading performance for the gray and red-on-green charts could also result from local luminous contrast artifacts in the red-on-green chart. These could arise from the small difference in luminous reflectance between the red and green colors, nonuniformities in the printing process, or chromatic aberration. The red-on-green chart has a residual Michelson luminous contrast of 0.8% (Weber contrast, 1.6%). This contrast level is too small by itself to equate the reading performance with the gray chart with a luminous contrast of 28% (Weber contrast, 77.8%).^25^^,^^26^ It is possible that the residual 0.8% achromatic contrast artifact could add to the chromatic contrast in the chart; however, based on the study by Knoblauch et al.^3^ showing that adding chromatic contrast to low contrast (<12% Michelson) achromatic contrast is additive, we would expect the red-on-green chart to perform in between the original and gray chart.^4^ Local luminance contrast artifacts within the lines of text, however, cannot be ruled out based on the normal data. That is the reason for further investigating the performance of individuals with congenital red–green color vision defects. If individuals with a selective loss of red–green chromatic sensitivity have similar reading performance on the red-on-green chart as on the gray chart, then sufficiently large luminous contrast artifacts may exist on the red-on-green chart, and that chart is therefore not measuring chromatic sensitivity but rather achromatic sensitivity. The alternative is that the difference in the S-cone response is contributing to the chromatic contrast.

Table 2 shows that the color defective reading acuities are lower and the CPSs are larger for the red-on-green chart relative to the gray chart, albeit the difference is small for two of the three anomalous trichromats. The differences between the red-on-green and gray charts for the deuteranope and the other anomalous trichromat are larger, approaching 0.1 logMAR. The results for the latter two participants, particularly the deuteranope, indicate that the luminous contrast or S-cone response artifacts on the red-on-green chart are minimal, at least for the smaller print sizes. One might expect the protanomalous red–green results to be similar to the gray chart because their reduced sensitivity to long wavelength light would produce luminous contrast artifacts where the red print is darker than the background. The small differences between the gray and red-on-green charts for the other deuteranomaly may have occurred because the red–green contrast on the chart was sufficiently above their threshold. The background and letter colors for the red-on-green chart were selected so that the red–green contrast was near the plateau of the reading speed–print size function for larger print based on the findings of Legge et al.^4^ for the 1° letter height. Based on the results of these two anomalous trichromats, the chromatic contrast level selected for the chart may be too large for color vision screening.

The choice of using text defined by red–green chromatic contrast was based on the earlier study by Legge et al.^4^ that provided data for setting our contrast values. The value for the L-/M-cone contrast was selected so that it was near the plateau of the reading speed–print size function for larger print based on the findings of Legge et al.^4^ for the 1° letter height. This reading level then sets the target value for the gray chart. Nevertheless, selecting a red–green chromatic chart seems contrary to the findings that blue–yellow acquired defects are more common in the older population^27^ than red–green defects. However, Legge et al.^4^ and other studies have reported that reading performance for red text on a dark background is impaired relative to other colored text of equal luminance in various low vision conditions, including inherited cone disorders,^15^ diabetic retinopathy, and optic atrophy,^14^ thus indicating that the red–green pathway can also be affected by the visual disorder. The impaired reading performance for the red text is likely due to the pseudoprotanomaly that often accompanies the type I red–green^28^ and type III blue–yellow acquired defects.^29^ Thus, comparing the red–green acuity to the gray and black-on-white BAL chart may be useful in diagnosing and managing a variety of visual disorders. Nevertheless, a chart with text defined by a blue–yellow chromatic contrast would also be helpful as part of the near chart test battery for assessing chromatic and achromatic reading performance. It is currently under development.

### Limitations of the Study

A limitation of this study is the lack of detailed refractive data for all participants. Although only 38% of participants had refractive error and wore their usual correction, we could not collect complete data for all subjects, which may affect the generalizability of our findings. The question remains whether the text on the printed red–green acuity chart is equal in luminance to the background for all positions on the card. Differences could arise from printing artifacts, chromatic aberration, or both. That the individuals with a severe red–green color defect had reduced RA and corresponding larger CPS indicates that the luminances are essentially equal for the smaller text. Nevertheless, the result that the MRS for the deuteranope was similar to the gray chart indicates that there could be possible luminous contrast artifacts at the larger print sizes. Other possibilities are that red–green colors were not on the same line of confusion for that individual or a combination of these two factors. In addition, we could not independently confirm that the chromaticities were uniform throughout the chart because of limitations in our equipment. These limitations raise the issue of how sensitive the red-on-green chart is to selective losses in red–green chromatic sensitivity. We also do not know how the chromatic near-point card used in a clinical setting applies to situations where people read near their MRS. These questions will be addressed in future studies.

Finally, the red–green near-point card requires a light with a correlated color temperature of at least 90 and a color rendering index of 6500K. Although these lamps are easier to obtain due to improvements in light-emitting diode (LED) lighting technology, the restrictions on the spectral quality of the lamp do constrain the clinical utility of the test.

## Conclusions

We have collected extensive data for a near visual performance chart consisting of words in contextual sentences presented using the Arabic language to better understand how color contrast influences reading. The chart can be used to measure acuity, reading speed, and CPS for text that is created using red–green chromatic contrast. Because the luminous reflectance of both the characters and the background is lower than the original high-contrast black-on-white BAL chart, the red-on-green chart was compared to a gray chart with a similar background luminance and lower contrast than the original chart. The major difference between the red-on-green and gray charts relative to the original BAL chart is that the RA for the two newer charts is poorer, and the CPS is larger than the original BAL chart. The MRS is similar for all three charts, confirming earlier findings that, if either luminous or chromatic contrast is sufficiently above threshold for a given size text, then the MRS is similar. For individuals with a selective loss, or greater loss, along the red–green chromatic axis, we would expect a relatively larger reduction in the red–green RA and a corresponding increase in the CPS relative to the gray chart. However, the MRSs approaching the levels found on both the gray and black-on-white charts indicated that there was a sufficient number of text sizes larger than the CPS on the red–green chart to determine the MRS.

## References

[1] Legge GE. *Psychophysics of Reading in Normal and Low Vision*. Boca Raton, FL: CRC Press; 2007.

[2] Carver RP. *Reading Rate: A Review of Research and Theory*. San Diego, CA: Academic Press; 1990.

[3] Knoblauch K, Arditi A, Szlyk J. Effects of chromatic and luminance contrast on reading. *J Opt Soc Am A*. 1991; 8: 428–439. 2007918 10.1364/josaa.8.000428

[4] Legge GE, Parish DH, Luebker A, Wurm LH. Psychophysics of reading. XI. Comparing color contrast and luminance contrast. *J Opt Soc Am A*. 1990; 7: 2002–2010. 2231110 10.1364/josaa.7.002002

[5] Eperjesi F, Fowler CW, Kempster AJ. Luminance and chromatic contrast effects on reading and object recognition in low vision: a review of the literature. *Ophthalmic Physiol Opt*. 1995; 15: 561–568. 8594527

[6] Dain SJ, Floyd RA, Elliot RT. Color and luminance increment thresholds in poor readers. *Vis Neurosci*. 2008; 25: 481–486. 18598422 10.1017/S0952523808080565

[7] Bouldoukian J, Wilkins AJ, Evans BJ. Randomised controlled trial of the effect of coloured overlays on the rate of reading of people with specific learning difficulties. *Ophthalmic Physiol Opt*. 2002; 22: 55–60. 11829008 10.1046/j.1475-1313.2002.00002.x

[8] Evans BJ, Cook A, Richards IL, Drasdo N. Effect of pattern glare and colored overlays on a stimulated-reading task in dyslexics and normal readers. *Optom Vis Sci*. 1994; 71: 619–628. 7877805 10.1097/00006324-199410000-00004

[9] Mullen KT. The contrast sensitivity of human colour vision to red-green and blue-yellow chromatic gratings. *J Physiol*. 1985; 359: 381–400. 3999044 10.1113/jphysiol.1985.sp015591 PMC1193381

[10] McLean MV. Brightness contrast, color contrast, and legibility. *Hum Factors*. 1965; 7: 521–526. 5886091 10.1177/001872086500700603

[11] Penkelink G, Besuijen J. Chromaticity contrast, luminance contrast, and legibility of text. *J Soc Info Disp*. 1996; 4: 135–144.

[12] Alabdulkader B, Leat SJ. A standardized Arabic reading acuity chart: the balsam alabdulkader-leat chart. *Optom Vis Sci*. 2017; 94: 807–816. 28737607 10.1097/OPX.0000000000001103

[13] Alsalem NI, Almustanyir A, Alhassan M, et al. Can chromatic text/background improve Arabic reading performance? *Saudi J Ophthalmol*. 2023; 37: 218–221. 38074308 10.4103/sjopt.sjopt_44_23 PMC10701150

[14] Jacobs KL, Leat SJ, Hovis JK. The effect of color on the reading performance of low vision patients. In: Dickinson C, Murray I, Carden D, eds. *John Dalton's Colour Vision Legacy*. London: Taylor & Francis; 1997: 207–213.

[15] Legge GE, Rubin GS. Psychophysics of reading. IV. Wavelength effects in normal and low vision. *J Opt Soc Am A*. 1986; 3: 40–51. 3950791 10.1364/josaa.3.000040

[16] Calabrèse A, Cheong AMY, Cheung S-H, et al. Baseline MNREAD measures for normally sighted subjects from childhood to old age. *Invest Ophthalmol Vis Sci*. 2016; 57: 3836–3843. 27442222 10.1167/iovs.16-19580 PMC4961000

[17] Pitt FG. The nature of normal trichromatic and dichromatic vision. *Proc R Soc Lond B Biol Sci*. 1944; 132: 101–117.

[18] Stokes M, Fairchild MD, Berns RS. Precision requirements for digital color reproduction. *ACM Trans Graph*. 1992; 11: 406–422.

[19] Alabdulkader B, Leat SJ. Toward developing a standardized Arabic continuous text reading chart. *J Optom*. 2017; 10: 84–94. 27162118 10.1016/j.optom.2016.03.003 PMC5383463

[20] Committee on Vision. Recommended standard procedures for the clinical measurement and specification of visual acuity. Report of Working Group 39. Committee on Vision. Assembly of Behavioral and Social Sciences, National Research Council, National Academy of Sciences, Washington, D.C. *Adv Ophthalmol*. 1980; 41: 103–148. 7001873

[21] Alabdulkader B, Almatar H, Alshubaili H, et al. Age-related changes in reading performance in normally sighted Arabic-speaking adults. *Clin Exp Optom*. 2024; 107: 806–812. 38402851 10.1080/08164622.2023.2298777

[22] Trauzettel-Klosinski S, Dietz K. Standardized assessment of reading performance: the new international reading speed texts IREST. *Invest Ophthalmol Vis Sci*. 2012; 53: 5452–5461. 22661485 10.1167/iovs.11-8284

[23] Lovie-Kitchin J, Brown B. Repeatability and intercorrelations of standard vision tests as a function of age. *Optom Vis Sci*. 2000; 77: 412–420. 10966067 10.1097/00006324-200008000-00008

[24] Bailey I, Clear R, Berman S. Size as a determinant of reading speed. *J Illum Eng Soc N Am*. 1993; 22: 102–117.

[25] Fujita K, Oda K, Watanabe J, Yuzawa M. How normal eyes perform in reading low-contrast texts. *Jpn J Ophthalmol*. 2008; 52: 44–47. 18369699 10.1007/s10384-007-0494-6

[26] Ohnishi M, Otsukuni T, Takahashi A, et al. Effects of luminance contrast and character size on reading speed. *Vision Res*. 2020; 166: 52–59. 31855668 10.1016/j.visres.2019.09.010

[27] Schneck ME, Haegerstrom-Portnoy G, Lott LA, Brabyn JA. Comparison of panel D-15 tests in a large older population. *Optom Vis Sci*. 2014; 91: 284–290. 24535417 10.1097/OPX.0000000000000152 PMC4014780

[28] Pokorny J, Smith VC, Ernest JT. Macular color vision defects: specialized psychophysical testing in acquired and hereditary chorioretinal diseases. *Int Ophthalmol Clin*. 1980; 20: 53–81. 6967472

[29] Verriest G. Further studies on acquired deficiency of color discrimination. *J Opt Soc Am*. 1963; 53: 185–195. 13996879 10.1364/josa.53.000185

